# How Does Nursing Staff Perceive the Use of Electronic Handover Reports? A Questionnaire-Based Study

**DOI:** 10.1155/2011/505426

**Published:** 2011-06-09

**Authors:** Torbjørg Meum, Gro Wangensteen, Karen S. Soleng, Rolf Wynn

**Affiliations:** ^1^Telemedicine Research Group, Department of Clinical Medicine, University of Tromsø, 9038 Tromsø, Norway; ^2^Tromsø Telemedicine Laboratory, University Hospital of North Norway, 9037 Tromsø, Norway; ^3^Divison of Addiction and Specialized Psychiatry, University Hospital of North Norway, 9291 Tromsø, Norway

## Abstract

Following the implementation of electronic nursing records in a psychogeriatric ward, we examined nursing staff's attitudes and perceptions to the implementation of an electronic handover routine. A web-based anonymous and secure questionnaire was distributed by e-mail to all nursing staff at a psychogeriatric ward at a university hospital. Most respondents were satisfied with the electronic handover, and they believed they managed to keep informed by the new routine. The simultaneous introduction of a morning meeting, to ensure a forum for oral professional discussion, was a success. A minority of staff did not fully trust the information conveyed in the electronic handover, and a significant proportion expressed a need for guidance in using the system. Staff that had a high level of trust in written reports believed these saved time, had little trouble finding time and a place to read the reports, and were more positive to the new handover routine.

## 1. Background

Having adequate and up-to-date information and knowledge about patients is crucial in order to maintain continuity of care [1, 2]. The traditional oral handover, where nurses orally give information and their assessment of the situation and needs of individual patients (supported by some brief written notes), has played a central role in securing that information has been exchanged adequately. The oral handover has become an important part of nursing practice and is strongly embedded in hospital culture [3]. However, it has become a well-known fact that hospitals and other parts of the health care service suffer from communication problems [4, 5]. Often, such problems are the cause of more serious medical and nursing errors [6, 7]. Although it clearly has some advantages, such as immediacy, the possibility for interactivity, and its social importance as a meeting point for nurses [8], the traditional oral handover has been identified as one possible area for miscommunication—for instance, because central information is not conveyed. Some, therefore, recommend a more standardized hand-off practice [9]. It has also been challenged as being ineffective [10], focusing on physical aspects of care [11], and that a majority of information present in the oral handover should be available in formal documentation sources [12]. Thus, it is not surprising that many nurses (22%–61%) have expressed dissatisfaction with the traditional oral handover [13].

The electronic patient record (EPR) has recently become a key factor in the information flow, and it has been suggested that using information technology can be an effective method for improving quality, efficiency, and costs [14–16]. In any case, it is evident that the introduction of the EPR reshapes information management, creates new communication patterns, and enables the development of new practice models in nursing [17]. Recently, the development of the profession as well as legal concerns have lead to an increased emphasis on nursing care plans and written nursing documentation. Some studies [18–21] have suggested that the introduction of the written handover procedure has improved nursing documentation as well as communication between nurses. The introduction of the electronic handover is quite recent, and few have examined how nursing staff have responded to this change in routines. Moreover, there have been relatively few studies of handover routines in nonacute psychiatric wards [22].

In the present study, we report on the attitudes and perceptions of nursing staff to the electronic handover routine at a ward in a Norwegian university hospital.

## 2. Methods

### 2.1. Study Ward

#### 2.1.1. Patients and Providers

The study was carried out at the Psychogeriatric Ward at the University Hospital of Northern Norway (UNN). The patients were 65 years or older and the hospitalization typically lasted 6–8 weeks. This is an inpatient ward with 14 beds that treats patients who are suffering from psychiatric disorders, including depression, psychosis, dementia, and anxiety disorders. The problems facing patients are complex, since most of the patients suffer from somatic illnesses in addition to the psychiatric illness. The patients are subjected to various types of tests (blood work, imaging, physical examination, psychological testing, etc.) and are diagnosed, and appropriate treatment is subsequently initiated. The work at the ward is highly interdisciplinary, and the clinical staff comprises psychiatrists, physicians, psychologists, nurses, assistant nurses, social workers, occupational therapists, physiotherapists, and some unskilled staff. The number of nursing staff on duty at the ward varies. Normally, there are 8 on the day shift, 6 on the evening shift, and 3 on the night shift. Nurses from all shifts were asked to participate in the study. Since the nursing work is based on primary nursing, that is, where a nursing team provides complete care for a small group of patients, the staff gets to know the patients well during the stay.

#### 2.1.2. Handover Routine and Electronic Nursing Documentation at the Ward

At the study ward, electronic nursing documentation was introduced in 2005. In 2008, a new handover routine was implemented. Instead of presenting all information orally (a 30 minute meeting at each handover covering all patients in the ward), staff would now read the relevant electronic nursing care plans regarding their own patients only. 

In addition, every morning, there was now a 30 minute meeting for professional discussion, usually covering 2-3 patients, but also including discussions not relating to specific patients. This type of meeting was only held during the morning shift. The main objective of the morning meeting was to provide an opportunity for discussion and reflection in relation to various nursing topics, and thus ensure the multifunctional aspect to the handover process [8]. During the meeting, the electronic care plan was visualized on the screen wall by a projector to provide an opportunity for collective reading and participation. The morning meetings were usually headed and facilitated by a professional development nurse. The topics of the meetings were not prearranged, and staff could suggest topics at the beginning of each meeting. Typically, discussions focused on matters related to current nursing challenges in the ward. Apart from some items in the questionnaire study reported here, there was no systematic evaluation of the morning meetings, but oral feedback from the participants was used to make minor adjustments to the form and content of the meetings.

Moreover, to facilitate the introduction of the new handover process and make a gradual transition to the new structure, it was decided to keep the oral handover on Monday mornings and Friday afternoons and also the oral handover between the evening and night shifts. The remaining traditional oral handovers were meant to supplement the new system where the nurses read the relevant electronic nursing care plans, and the nurses were required to read up on their own patients also when traditional oral handover meetings were arranged.

Use of the new electronic system also required skills and knowledge of the use of the care plan in general, as well as the use of a standardized language. At the core of the nursing care plan was its shared terminology to describe the patient's problem (i.e., nursing diagnosis) and link this to one or more interventions. The classification systems of the North American Nursing Diagnosis Association (NANDA) and the Nursing Intervention Classification (NIC) were embedded in the system and had become an integrated part of the electronic documentation of nursing at the ward [23]. 

To secure the quality of the electronic nursing care plans during their implementation phase, support and supervision was offered by two specially trained nurses. They were available every day between 12 noon and 1.30 p.m. and could assist the other nursing staff in making sure that the nursing documentation was reliable and updated. The guidance was focused on nursing care plans in general and on use of the electronic records system.

### 2.2. The Questionnaire

A questionnaire was designed in order to examine the attitudes and perceptions of the nursing staff to the new electronic handover practice. The questionnaire focused on issues related to perceived usefulness and perceived ease of use and was inspired by the technology acceptance model (TAM) [24]. TAM is a theory adapted from the theory of reasoned action [24] and was tailored for modeling user acceptance of information technology. According to TAM theory, two determinants are particularly important for users' acceptance: perceived usefulness and perceived ease of use. Several developments and extensions to TAM have been proposed, typically including several determinants of intention and usage and also moderators of effects [25–27]. The main idea in TAM and its later modifications, that is, the relationship between perceived usefulness and intention to use/actual use, is simple and appears to stand robust [28].

Some of the items in the questionnaire used in the present study resembled items on the 6+6 item versions of the TAM questionnaires originally described by Davis [24], while other items were more specific to the circumstances of the study ward. The appropriateness of the questionnaire-items was discussed in a small group of project members, including clinicians, in order to increase the face validity of the questionnaire. Two of the group members (nurses that had working experience from the study ward) made a first version of the questionnaire, and two other group members (a nurse and a doctor, both with clinical and research experience) gave comments and suggestions; this process was repeated several times until the complete group was satisfied with the final result. 

The questionnaire was sent to all nursing staff in the form of an e-mail link to a secure web-based form. The researchers did not have access to information regarding who had responded to the questionnaire (and who had not). The questionnaire did not contain information that could be used to identify individual respondents, and all respondents were anonymous. The questionnaire consisted of 22 questions and covered different topics, including questions on satisfaction with the new handover practice and the perceived effectiveness and usefulness of the EPR system. There were also questions about how the respondents felt about the new morning meeting and questions relating to guidance and support with respect to the new handover routine as well as the EPR in general. Three possible options were provided to each question, with the response options low, medium, and high. The questionnaire also included an open commentary field. In the questionnaire, there was also information about the purpose of the survey, information stating that participation was voluntary, and that anonymity and confidentiality were assured. The study was approved by the Head of the Hospital Division and was in accordance with the regulations of the Hospital's Data Protection Officer. The survey sampled anonymous information about staff attitudes, and no patient information was sampled.

### 2.3. Statistical Procedures

Data were analysed descriptively. In addition, a multiple regression analysis was performed in order to examine predictors of satisfaction with the new handover routine. Items entered as predictors in the model included whether the respondents found time and place to read, their trust in written documentation, whether they believed reading saved time, whether they believed other written sources were important, whether they believed morning meetings were important, whether they believed working on handover routines improved nursing standards, whether they felt independent in writing nursing documentation, and if they believed guidance was important in implementing the new routine. The results were analyzed using the statistics software SPSS 16.0. A significance level of *P* < .05 was employed.

## 3. Results

### 3.1. The Sample

Of the 34 that were eligible to participate, 32 responded, giving a response rate of 94%. 80% of the respondents had more than two years of work experience.

### 3.2. The Electronic Handover

Seventy-five percent were very satisfied or satisfied with the electronic handover (Table 1), and 78% also felt the system was satisfactory in securing that they were updated on the patients' situation and needs. Most staff (93.8%) were also satisfied or very satisfied with making their own assessments regarding the need for information about specific patients.

In this study, we did not ask which information sources they used, but earlier investigations at the ward have shown that the nursing care module in the EPR system has become the single most important element in the documentation work of the nursing staff [22]. Nevertheless, there was still a certain amount of skepticism in trusting electronic nursing notes, and only 37.5% felt they could rely unconditionally on the nursing module in the EPR. Informal sources of information, such as the weekend summary and the message book, still play an important role in the information exchange, and 93.5% responded that such sources of information were very important or important. 

### 3.3. The Morning Meeting

The other new intervention in the new handover process was the introduction of the morning meeting, and all the respondents felt that the morning meeting was important or very important to professional development, and they were also satisfied or very satisfied with the topics that were discussed during the meetings.

### 3.4. Guidance

Interestingly, almost all (96.2%) the respondents considered the daily documentation guidance significant to their professional development. Although they had been using EPR and the electronic nursing module for several years, there were still many who needed guidance to use the EPR, to find nursing concepts, and to make changes in the care plans, and 15.4% felt they needed a lot of guidance.

### 3.5. Predictors of Satisfaction with New Handover Routine

A multiple regression analysis was performed, where a set of variables (see Table 2) were entered as predictors and degree of satisfaction with the new handover routine was the outcome variable. In the analysis, we found that staff that were able to find a time and place to read the written (electronic) report, that expressed a high level of trust in written (electronic) reports, that believed written (electronic) reports saved time, that considered other sources of information as less important (i.e., message book, weekend summary, etc.), and that believed that working on handover routines improved nursing standards were significantly more satisfied with the use of the electronic handover than other staff. We also found that being less content with the topics of the morning meeting and having less faith in the importance of the oral handover were related to a higher degree of satisfaction with the new handover routine. Believing that one was independent in writing nursing documentation or expressing that guidance in using the nursing documentation was important were not significant predictors (Table 2). Thus, the results may be taken to suggest that nursing staff that felt they had sufficient opportunity to use the electronic nursing record, and those who trusted the electronic records and felt they saved time were generally more satisfied with the new handover routine. Believing that improving routines was important and having less faith in the traditional oral handover were also associated with satisfaction with the new handover routine. 

## 4. Discussion

The main finding in this study is that the nursing staff was satisfied with the electronic handover routine. They were content with being able to judge which information they needed and believed staff were kept updated on patients' needs. However, the implementation of the new electronic handover procedure has involved a paradigm shift in the information work at the ward; the structure of the handover has changed, and the focus has moved from oral to written information. The successful introduction of the new electronic handover is composed by multiple factors that provide new opportunities but also involve new challenges. 

The study has showed that some nursing staff need guidance in order to make use of the electronic nursing documentation system, including the electronic handover. Such a system for guidance should be in place in order to secure a smooth transition from an oral to an electronic handover practice. The study has demonstrated that a gradual implementation of a new handover process has been adopted by the users, and it is likely that one reason for a successful adoption was that local and professional needs were ensured [29].

The use of electronic care plans and nursing classifications has become a major factor in the information work at the ward, and our study shows that a majority of nursing staff rely on the written documentation. Reliable and accurate documentation was a prerequisite for changing the handover process. Before the implementation of the electronic nursing module, the documentation was incomplete, and the language used in the documentation was inaccurate and often included local (i.e., to the ward) jargon, as has been shown to be the case in other wards [11]. The use of the care plans has increased significantly after the implementation of the nursing module and provides a more complete documentation of both given and planned care [22]. As a main source of information and knowledge of the patients, the use of the electronic nursing module provides a good structure for information exchange between nursing staff and provides a standardized language that contributes to a common understanding of given and planned care. Our findings are also in accordance with legal, political, and professional strategies to electronic cooperation in the health and social sector and are consistent with similar studies [18–20]. However, there are still some that are not satisfied with the new handover routines and some that do not trust the written documentation. This tension between written documentation and face-to-face communication is well described in the nursing literature [30] and in health informatics [31]. Clinical judgment in tremendously complex, requires various types of knowledge, and involves both explicit and tacit knowledge. The tacit, embedded know-how is gained from experience and is interplay between theoretical knowledge and practical know-how. This kind of knowledge is difficult to formalize in an electronic care plan, and some kind of face-to-face communication is necessary to ensure all aspects of clinical judgment and reflection. With the introduction of the new morning meeting, this kind of face-to-face communication is ensured. As the shifts of the nursing staff rotate, all nursing staff get the opportunity to participate (although not all the time).

The study also shows that all were satisfied with the topics and the impact of the morning meetings on professional development. Typical topics for the meetings were different problems related to care issues, and the meetings provided an opportunity for collective reflection and the development of clinical judgment [30]. It is usually the nurse in charge of professional development that leads the meeting, but the topics are determined in collaboration with nursing staff. Often, it is 2-3 patient cases that form the basis of the discussion. Use of the electronic care plans has also become a key element during the meetings, and they are used as a basis for discussion and reflection. Since the care plans are visualized by projecting the PC screen on the wall in the conference room, they provide an opportunity for collective reading and discussion. This interaction provides an opportunity to socialize, share, and learn from each other's experiences as well as an opportunity for emotional support. 

The regression analysis demonstrated several predictors of satisfaction with the new handover routine. Particularly interesting was the finding that those who had a high level of trust in written reports, believed these saved time, and had little trouble finding time and a place to read the reports were more positive to the new handover routine. While the small sample of the present study should be taken into consideration when analyzing the results, this finding could suggest that the successful (in terms of the satisfaction of the nursing staff) implementation of electronic nursing routines in part depends on nursing staff being used to written reports. Extrapolating from this idea, one can hypothesize/speculate that wards that to a lesser extent have used written (i.e., pen and paper) reports will need more time and effort to successfully implement new electronic routines. 

There were limitations to the study. Although the response rate was quite high, the sample was relatively small and covered one ward only. Thus, it is problematic to claim that the findings of the present study reflect nurses' acceptance of electronic handovers in general. Studies with larger samples will be needed to verify the findings of the present study and to make more general claims about nurses' attitudes to electronic handovers. Moreover, several changes in ward routines were simultaneously introduced, that is, the new (electronic) handover routine and the new morning meeting. Although we believe it was necessary to make both changes at the same time, we do not know how satisfied the nursing staff would have been with the new handover routine without the new morning meeting.

This study contributes to an increased understanding of the nursing handover per se and to the implementation of an e-health solution in clinical practice. Further studies should address implications of the electronic handover for issues such as the impact on the quality of patients' care, patients' satisfaction, and the impact on how information is shared within and across organizational boundaries.

## 5. Conclusions

Most of the nursing staff was satisfied with the electronic handover procedure although a minority was less trustful of written information. Introducing a forum for oral discussion on topics of importance to the staff's professional development and securing sufficient guidance on how to use the system were perceived as central factors to success in the implementation. 

## Figures and Tables

**Table 1:** The electronic handover.

Item	High % (*n*)	Med. % (*n*)	Low % (*n*)
How satisfied are you with the electronic handover as a source of information?	18.8 (6)	56.2 (18)	25.0 (8)
How satisfied are you with making your own assessment regarding need for patient info?	18.8 (6)	75.0 (24)	6.2 (2)
Do you manage to keep informed in the new handover process?	12.5 (4)	65.6 (21)	21.9 (7)
Can you rely on the written information that you read in the electronic care plan and nursing notes?	37.5 (12)	59.4 (19)	3.1 (1)
How important are other sources of information like the message book and weekend summary?	67.7 (21)	25.8 (8)	6.5% (2)*

*Response missing on this item.

**Table 2:** Predictors of satisfaction with new handover routine.

Predictor	*B*	SE	Standardized *B*	*P*
Finds time and place to read	.363	.115	.446	**.006**
Trusts written documentation	.429	.191	.349	**.038**
Believes reading saves time	.312	.146	.314	**.046**
Believes other written sources are important	−.340	.123	−.332	**.013**
Believes morning meetings are important	.481	.178	.470	**.014**
Is content with topics in morning meeting	−.577	.195	−.609	**.009**
Believes having two oral handovers a week is important	−.341	.158	−.310	**.045**
Believes working on handover routine improves nursing standards	.342	.157	.361	**.043**
Independent in writing nursing documentation	.231	.128	.326	.087
Believes guidance is important	−.330	.166	−.401	.063
